# Medication-Related Osteonecrosis of Jaws (MRONJ) Prevention and Diagnosis: Italian Consensus Update 2020

**DOI:** 10.3390/ijerph17165998

**Published:** 2020-08-18

**Authors:** Giuseppina Campisi, Rodolfo Mauceri, Francesco Bertoldo, Giordana Bettini, Matteo Biasotto, Giuseppe Colella, Ugo Consolo, Olga Di Fede, Gianfranco Favia, Vittorio Fusco, Mario Gabriele, Antonio Lo Casto, Lorenzo Lo Muzio, Antonia Marcianò, Marco Mascitti, Marco Meleti, Michele D. Mignogna, Giacomo Oteri, Vera Panzarella, Umberto Romeo, Andrea Santarelli, Paolo Vescovi, Claudio Marchetti, Alberto Bedogni

**Affiliations:** 1 Department of Surgical, Oncological, and Oral Sciences, University of Palermo, 90127 Palermo, Italy; campisi@odonto.unipa.it (G.C.); odifede@odonto.unipa.it (O.D.F.); antonio.locasto@unipa.it (A.L.C.); panzarella@odonto.unipa.it (V.P.); 2 IAC-ONJ (Italian Allied Committee on ONJ), Temporary Chair at University of Palermo, 90100 Piazza Marina, Italy; francesco.bertoldo@univr.it (F.B.); giordanabettini@gmail.com (G.B.); fusco.dott.vittorio@gmail.com (V.F.); paolo.vescovi@unipr.it (P.V.); claudio.marchetti@unibo.it (C.M.); alberto.bedogni@unipd.it (A.B.); 3 Department of Biomedical and Dental Sciences and Morphofunctional Imaging, University of Messina, 98124 Messina, Italy; antoniam@hotmail.it (A.M.); oterig@unime.it (G.O.); 4 Department of Medicine, University of Verona, 37134 Verona, Italy; 5 Regional Center for Prevention, Diagnosis and Treatment of Medication and Radiation-Related Bone Diseases of the Head and Neck, University of Padova, 35128 Padova, Italy; 6 Department of Medical, Surgical and Health Sciences, University of Trieste, 34129 Trieste, Italy; m.biasotto@fmc.units.it; 7 Department of Medical, Surgical and Dental Speciality, University of Campania “Luigi Vanvitelli”, 80138 Naples, Italy; giuseppe.colella@unicampania.it; 8 Department of Surgery, Medicine, Dentistry and Morphological Sciences with Transplant Surgery, Oncology and Regenerative Medicine Relevance, University of Modena and Reggio Emilia, 41125 Modena, Italy; ugo.consolo@unimore.it; 9 Department of Interdisciplinary Medicine, University of Bari Aldo Moro, 70124 Bari, Italy; prof.gfavia@gmail.com; 10 Azienda Ospedaliera SS. Antonio e Biagio e Cesare Arrigo, Azienda Ospedaliera Nazionale SS. Antonio e Biagio e Cesare Arrigo, 15121 Alessandria, Italy; 11 Department of Surgical, Medical and Molecular Pathology and Critical Care Medicine, University of Pisa, 56126 Pisa, Italy; mario.gabriele@med.unipi.it; 12 Department of Clinical and Experimental Medicine, University of Foggia, 71121 Foggia, Italy; lorenzo.lomuzio@unifg.it; 13 Department of Clinical Specialistic and Dental Sciences, Marche Polytechnic University, 60126 Ancona, Italy; MarcoMascitti86@hotmail.it (M.M.); andrea.santarelli@staff.univpm.it (A.S.); 14 Department of Medicine and Surgery, University of Parma, 43125 Parma, Italy; marco.meleti@unipr.it; 15 Department of Neuroscience, Reproductive and Odontostomatological Sciences, University of Naples Federico II, 80131 Napoli, Italy; mignogna@unina.it; 16 Department of Oral and Maxillofacial Sciences, Sapienza University of Rome, 00161 Roma, Italy; umberto.romeo@uniroma1.it; 17 Department of Biomedical and Neuromotor Sciences, University of Bologna, 40125 Bologna, Italy

**Keywords:** MRONJ, prevention models, dentistry, oral surgery, bisphosphonates, denosumab, antiresorptive drugs, antiangiogenic agents, cancer, osteoporosis

## Abstract

The Medication-Related Osteonecrosis of Jaws (MRONJ) diagnosis process and its prevention play a role of great and rising importance, not only on the Quality of Life (QoL) of patients, but also on the decision-making process by the majority of dentists and oral surgeons involved in MRONJ prevention (primary and secondary). The present paper reports the update of the conclusions from the Consensus Conference—held at the Symposium of the Italian Society of Oral Pathology and Medicine (SIPMO) (20 October 2018, Ancona, Italy)—after the newest recommendations (2020) on MRONJ were published by two scientific societies (Italian Societies of Maxillofacial Surgery and Oral Pathology and Medicine, SICMF and SIPMO), written on the inputs of the experts of the Italian Allied Committee on ONJ (IAC-ONJ). The conference focused on the topic of MRONJ, and in particular on the common practices at risk of inappropriateness in MRONJ diagnosis and therapy, as well as on MRONJ prevention and the dental management of patients at risk of MRONJ. It is a matter of cancer and osteometabolic patients that are at risk since being exposed to several drugs with antiresorptive (i.e., bisphosphonates and denosumab) or, more recently, antiangiogenic activities. At the same time, the Conference traced for dentists and oral surgeons some easy applicable indications and procedures to reduce MRONJ onset risk and to diagnose it early. Continuous updating on these issues, so important for the patient community, is recommended.

## 1. Introduction

Medication-Related Osteonecrosis of Jaws (MRONJ) and its prevention play a role of great and rising importance, not only on the Quality of Life (QoL) of patients, but also on the decision-making process of the majority of dentists and oral surgeons, involved every day in MRONJ prevention (primary and secondary). The present report illustrates the update of conclusions adopted from the Consensus Conference held at the Symposium of the Italian Society of Oral Pathology and Medicine (SIPMO) on 20 October 2018, in Ancona (IT), when opinions were discussed and synthesized by 24 Italian experts. The Conference was the first national consensus to address the MRONJ disease and specific interventions [1,2,3], it shared the definition of MRONJ as an “adverse drug reaction described as the progressive destruction and death of bone that affects the mandible and maxilla of patients exposed to the treatment with medications known to increase the risk of disease, in the absence of a previous radiation treatment” [4], to be diagnosed and scored by clinics and radiographs [5], independently from the presence of exposed necrotic bone or bone probing via sinus/fistula tracts for more than 8 weeks [6,7,8,9,10,11].

The present report was considered by the experts as convenient and worthwhile in the light of the new findings [10,12,13] and the publication, in June 2020, of the new recommendations (2020) on MRONJ by SICMF/SIPMO [14], written on the inputs of the experts of the Italian Consensus group on ONJ (IAC-ONJ). Some good practices were confirmed, while others were partially or fully updated (e.g., a drug holiday in osteometabolic patients, see Section 2.5); additionally, figures and tables have been added to some issues to emphasize some of the good practices.

The updated Consensus Conference focused on the common practices still at risk of inappropriateness in MRONJ diagnosis and prevention, and on the dental management of patients exposed to ONJ. It is matter for the cancer (principally) and osteometabolic patients, since they are exposed to an increasing number of drugs with antiresorptive (i.e., bisphosphonates and denosumab) or antiangiogenic activities (e.g., bevacizumab; aflibercept; inhibitors of Tyrosine Kinases, TKIs; and mTOR inhibitors) [6,8,11,15,16,17,18]. At the same time, the authors updated the easy applicable indications and procedures for dentists and oral surgeons, in order to reduce MRONJ onset risk and to diagnose it early [14,19].

The experts focused on the topic of MRONJ using the following 6 main subcategories (issues): (1) clinical diagnosis; (2) radiologic diagnosis; (3) primary prevention (before and during drug intake); (4) dental management; (5) drug holiday (temporary suspension vs. therapeutic suspension); and (6) therapy.

The IAC-ONJ planned that a recommended practice equivalent will be advised for any inappropriate practice, within these 6 issues.

## 2. The Six Issues

### 2.1. Clinical Diagnosis of MRONJ: Practices at Risk of Inappropriateness and Good Practices

Assume that the Italian Consensus adopted the previously described definition of MRONJ [4,5], as being diagnosed and scored by clinics and appropriate imaging; besides the presence of only exposed necrotic bone or bone probing via sinus/fistula tracts for more than 8 weeks [7,8,9,10,11,20,21], the following practices for diagnosis were considered at risk of inappropriateness.

#### 2.1.1. Questionable Practices

(#1) Restricting the anamnestic interview only to the assumption of bisphosphonates for diagnosing MRONJ.

(#2) Carrying out routine bone biopsies when suspecting an MRONJ.

(#3) Considering the presence of exposed necrotic bone, in the oral cavity, as an essential (conditio sine qua non) sign to diagnose MRONJ.

(#4) Judging the presence of pain as an essential symptom for diagnosing MRONJ.

(#5) Believing that all MRONJ are preceded by dental invasive procedures.

#### 2.1.2. Good Practices

(#1) Evaluate not only the intake of bisphosphonate drugs (current or past), but also further pharmacological therapies (e.g., other antiresorptive agents, or drugs with anti-angiogenic activity) and perform a thorough physical examination and medical history, together with targeted radiologic examinations (see Section 2.2) (confirmed).

(#2) Perform jaw bone biopsies only if there is a suspicion of metastases in cancer patients (confirmed).

(#3) Take into the account not only the presence of exposed necrotic bone but considering also other clinical signs and first/second-level imaging (confirmed).

(#4) Consider that the symptom “pain” may not always be present in MRONJ cases, especially in the early stages (confirmed).

(#5) Consider that some cases of MRONJ can arise from the presence of dental-periodontal diseases or spontaneously, without any relation to invasive dental procedures (confirmed).

### 2.2. Radiologic Diagnosis of MRONJ: Practices at Risk of Inappropriateness and Good Practices

Radiologic imaging is assumed to be a fundamental tool for diagnostic completion, defining the extent of the disease at the skeletal level, staging and the correct therapeutic planning. Even if it is well known that no specific radiological signs of disease can be defined until now, an increasing volume of evidence recognizes some radiological characteristics that, although non-specific, are associated with MRONJ [6,9,13,22,23,24,25]. The use of radiological procedures must be aimed at the diagnostic/therapeutic advantage, in order to reduce the high social and biological costs deriving from radiation exposure [21]. MRONJ is definitely a bone disease that frequently affects the soft tissues, so it requires targeted imaging methods for diagnostic definition (first- and second-level exams) [13,26]. Only in cases of doubtful diagnosis may it be useful to resort to additional investigations, such as Magnetic Resonance (MR) and Nuclear Medicine tools (third-level exams), unless they are to be performed to monitor neoplastic disease, and not specifically prescribed for an MRONJ diagnosis.

#### 2.2.1. Questionable Practices

(#1) Starting therapies at risk of ONJ without a radiologic assessment of dental arches, remarkably in cancer patients.

(#2) Omitting the radiologic assessment for those patients at risk of MRONJ in the presence of dental/endo-periodontal diseases.

(#3) Prescribing to everybody exposed to therapies at risk of ONJ all imaging techniques to rule out MRONJ.

(#4) Requiring radiologic exams without a specific diagnostic hypothesis.

(#5) Prescribing radiological exams for MRONJ only after onset of jawbone exposure.

(#6) Diagnosing MRONJ only on the basis of the clinical signs, without radiologic assessment.

(#7) Prescribing high-resolution imaging for soft tissue (with contrast medium) for diagnosis of MRONJ.

(#8) Planning MRONJ treatment without second-level imaging exams for assessing jawbone disease extension and severity.

(#9) Monitoring the extension of the MRONJ process only by investigations of first-level exams (e.g., panoramic X-ray).

#### 2.2.2. Good Practices

(#1) Evaluate clinically and radiologically any local risk factors for MRONJ (i.e., dental/endo-periodontal diseases) for preventive dental screening in patients, remarkably cancer ones, who are candidates for therapies at higher risk of MRONJ; sometimes, MRONJ could manifest like a dental/endo-periodontal disease, or a worsening of a pre-existent disease (updated) [12].

(#2) Perform radiological exams in all cases of dental/endo-periodontal diseases in patients at risk of MRONJ (confirmed).

(#3) Reserve the indication for the execution of radiological exams of the second level (e.g., Computed Tomography, CT) to patients at MRONJ risk only in the presence of ascertained clinical or radiologic signs compatible with MRONJ (confirmed) [6,9,22].

(#4) Specify always the diagnostic hypothesis when prescribing radiological exams of any level (X-ray panoramic, CT) in patients at MRONJ risk (confirmed).

(#5) Do not delay in prescribing radiologic exams to investigate any clinical signs of possible MRONJ, independent of the presence of bone exposure or fistulas (updated).

(#6) Integrate always the clinical check-up with the appropriate imaging exams for the diagnosis of MRONJ (confirmed).

(#7) Prescribe radiologic exams for bone diseases without contrast medium for diagnosis of MRONJ (confirmed).

(#8) Set up the MRONJ treatment after defining its extension and severity also by targeted imaging (confirmed).

(#9) Use targeted imaging (i.e., CT every 6 months) to monitor MRONJ during follow-up after conservative and/or surgical treatment (updated).

### 2.3. Prevention of MRONJ: Practices at Risk of Inappropriateness and Good Practices

Assuming that recently it has been confirmed that MRONJ prevention means applying the correct protocols of (1) primary prevention for both pre-treatment and in-treatment patients, and (2) secondary prevention (i.e., early diagnosis of MRONJ) [5], the Consensus Conference believes that these principles should be followed by key figures such as physicians, drug prescribers, dentists and oral hygienists.

Primary prevention for MRONJ mainly means elimination/reduction of the oral and dental risk factors [5,11,15,27,28,29]; it is targeted at restoring and/or maintaining good oral health and reducing the risk of onset of pathological conditions or any other negative event. This approach has the greatest impact when aimed to constantly take care of oral health in patient at risk of MRONJ. Initially, about sixteen years ago, MRONJ (at that time labelled ONJ or BRONJ) was thought to be associated with dental extractions (or dentoalveolar surgery) in patients already being treated with bisphosphonates. More recently, infections at the dental-periodontal and peri-implant sites has been underlined as being one of the main local risk factors of developing MRONJ [12]. Intriguingly, these infections are often the main reason for surgical procedures of dental extraction or implant removal during or after therapy. It is advisable to consult the newest recommendations (2020) on MRONJ published by the two Italian scientific societies SICMF/SIPMO for a complete list of systemic and local risk factors [14] (Table 1).

A new emerging and huge aspect is that primary prevention should be performed not only prior to taking the MRONJ-related drugs but also during and after the treatment with antiresorptive agents (AR), in order to eliminate any infective outbreaks of MRONJ. It is the responsibility of the dentist to accurately assess the risk factors leading to the development of MRONJ and suggest a strategy to remove these factors or minimize the risk. The dentist must also stress the importance of maintaining effective dental hygiene (together with dental hygienist), including regular check-ups, for the patient. Both are necessary to maintain oral health, reducing the outbreak of MRONJ and/or detecting possible signs of the early symptoms of this disease.

Secondary prevention or early diagnosis is the second pillar in the strategy against MRONJ, since we know that MRONJ diagnosed at an early stage is more likely to be treated successfully. With respect to the diagnostic work-up, the main problem is the possible under-diagnosing of MRONJ cases without bone exposure (or fistula). A recent multicenter study [22,30] has demonstrated that the use of the traditional AAOMS [31,32] and ASBMR case definitions [33] may result in up to a quarter of undiagnosed BRONJ cases [34,35,36]. Thus, in order to avoid any underestimation of MRONJ, care should be taken with respect to the definition adopted by the Italian societies and to the use of the imaging studies in both cancer and osteometabolic patients, who are already receiving MRONJ-associated therapies, in combination or alone [8,28].

#### 2.3.1. Questionable Practices

(#1) Keeping teeth with endodontic and/or periodontal diseases at uncertain prognosis only to avoid extractive and invasive procedures, in patients at risk of MRONJ.

(#2) Extracting teeth in partial or total inclusion for pure preventive purposes in patients that are candidates for treatment with drugs at risk of inducing MRONJ.

(#3) Scheduling in cancer patients the first dental check-up only after diagnosis of bone metastases.

(#4) Omitting in osteometabolic patients dental check-ups for oral health status assessment and relative preventive dentistry actions.

(#5) Omitting periodic dental check-ups for edentulous patients or wearing removable prostheses, if they are at risk of MRONJ.

(#6) Underestimating the usefulness of the imaging performed to evaluate cancer disease extension (i.e., bone scintigraphy; CT-PET) with respect to MRONJ early diagnosis (and follow-up).

#### 2.3.2. Good Practices

**Figure 1:**
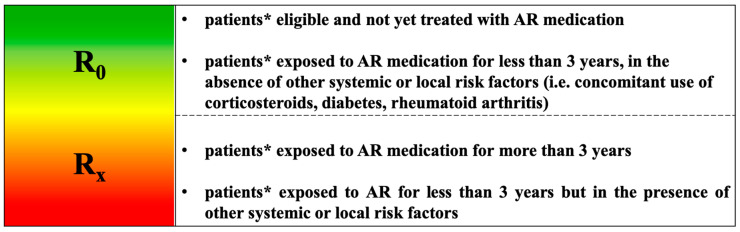
MRONJ risk profile of patients with metabolic bone fragility receiving AR medications [19]. * It includes also CTIBL patients (hormone deprivation therapy induced bone loss in breast and prostate cancer patients without bone metastases).

(#1) Extract teeth with endodontic and/or periodontal diseases, in patients at risk of MRONJ, when a conservative approach is not possible and a good tooth prognosis is not guaranteed (confirmed).

(#2) Maintain teeth in partial or total inclusion, if no sign and/or symptom is present (e.g., dysodontiasis, pericoronitis, decay, periodontal disease, caries and/or root resorption of the second molar, and mechanical trauma on the surrounding mucous membranes) (confirmed).

(#3) Schedule in cancer patients the first dental check-up as soon as possible, preferably during the tumor staging process (confirmed).

(#4) Consider in osteometabolic patients (generally at lower risk of MRONJ than cancer patients) a potential gradient in MRONJ risk (Figure 1) (updated) [19].

**Figure 2:**
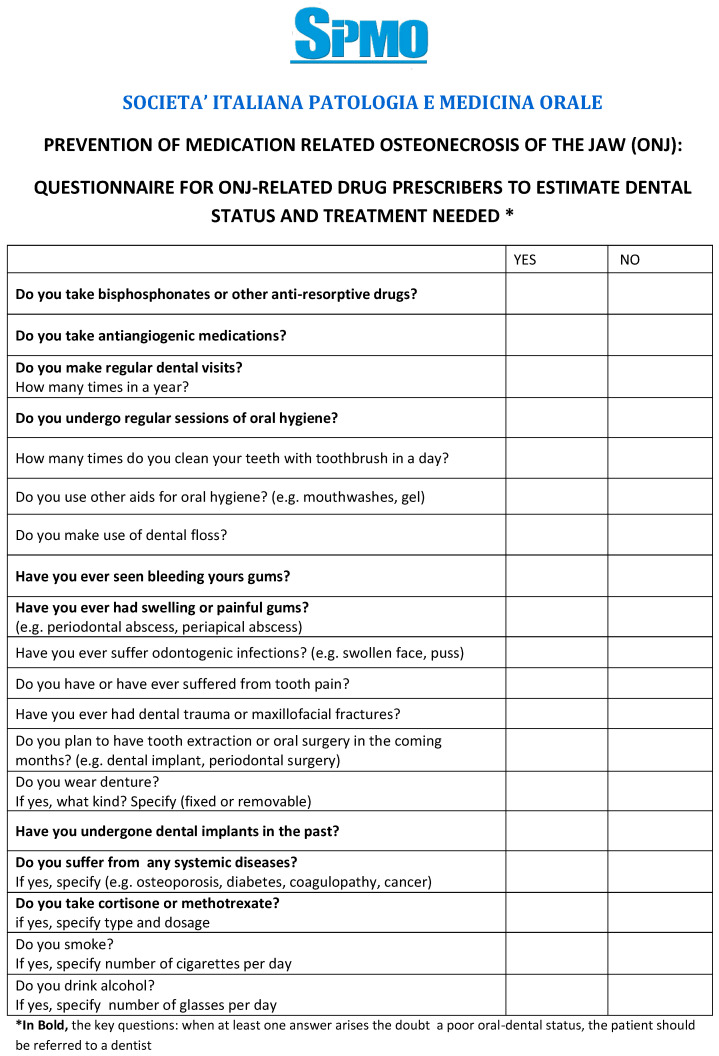
Questionnaire for ONJ-related drug prescribers.

(a) Administration and duration of treatment with MRONJ-related drugs [5,6,37,38]; consider in this category cancer patients receiving antiresorptive agents for the prevention of osteoporosis induced by hormone therapy (Cancer Treatment Induced Bone Loss, CTIBL), with a risk of MRONJ being considerably lower than that of cancer patients receiving AR drugs for bone metastases or myeloma;

(b) Eventual presence of comorbidities and further medical therapies indirectly associated; assess, during the prescription of the MRONJ-associated drugs, the oral health status even by a questionnaire (bypassing the preliminary dental check-up) (Figure 2) [39]; schedule semi-annual dental visits (the first one within 6 months from the first assumption of antiresorptive drugs).

(#5) Schedule preventive dental check-ups even in patients at risk of MRONJ even if edentulous or wearing removable dentures, in order to intercept and correct any injury on the oral mucosa (confirmed).

(#6) Research any sign of jawbone alterations in cancer patients, revising the pictures of the imaging investigations (e.g., bone scintigraphy, CT-PET) already performed to stage the cancer disease, in order to potentially detect eventual early signs of MRONJ, before prescribing further specific imaging (updated).

### 2.4. Dental Management: Practices at Risk of Inappropriateness and Good Practices

Assume that the Consensus Conference has now adopted the new protocols for dental management in patients at risk of MRONJ, published in the recommendations on MRONJ by the two scientific societies SICMF/SIPMO [14], in which it re-evaluated its opinion on the following hot points: (a) implants in cancer and osteometabolic patients; (b) antibiotic prophylaxis; and (c) healing for first vs. second intention.

#### 2.4.1. Questionable Practices

(#1) Building an implant rehabilitation in cancer patients who have to take or are taking MRONJ-associated drugs due to advanced cancer disease.

(#2) Considering as contraindicated, in an absolute sense, implant rehabilitation in osteometabolic patients taking MRONJ-associated drugs (including cancer patients treated with antiresorptive agents for CTIBL).

(#3) Prescribing antibiotic prophylaxis before non-invasive dental therapies.

(#4) Performing extractive procedures without removing, even if necessary, the alveolar bone, and plan the healing as a second intention (especially with cortisone administration).

#### 2.4.2. Good Practices

(#1) Avoid in cancer patients implant rehabilitation and plan non-surgical rehabilitation (confirmed).

(#2) Plan implant rehabilitation in osteometabolic patients taking into account the cumulative dose of the drug already taken, the periodontal status, lifestyle (e.g., smoking), comorbidities and other drugs. Share with the patient a hypothetical medium to long-term MRONJ risk due to “implant presence-triggers” (such as peri-implantitis and micro-cracks), beside the lower probable short-term risk due to “implant surgery-triggers” [29,40,41,42,43]. Plan informed consent and semi-annual follow-ups (updated).

(#3) Recommend always, in patients at MRONJ risk, antibiotic prophylaxis when surgical procedures involving the dento-alveolar process or maxillary bones are planned (e.g., extractions, periodontal/endodontic surgery, implants and pre-implant surgery) (confirmed).

(#4) Practice extractive or surgical procedures planning the healing as the first intention with the clot maintenance (confirmed).

### 2.5. Drug Holiday or Temporary Suspension for Patients at Risk of MRONJ: Practices at Risk of Inappropriateness and Good Practices

Assuming that a drug holiday is a controversial issue, involving both drug prescribers (oncologists, hematologists, bone health specialists and general practitioners) and oral and dental specialists, it is important to clarify that “Drug holiday” can have at least two meanings in the medical literature when referred to MRONJ. It can mainly mean (1) the temporary and preventative suspension of drug therapy (i.e., bisphosphonates, denosumab and sometimes antiangiogenic drugs) in a patient at risk of MRONJ, before the necessary dental procedures (tooth extraction or jawbone surgery); or (2) the suspension of drug therapy (i.e., bisphosphonates, denosumab) due to a diagnosis of MRONJ, hypothetically finalized to stop or to slow the osteonecrosis process (a practice with scarce literature as support). This document will afford only the first meaning of a “drug holiday”.

It is well known that suspension of drug therapy can create different cost–benefit balances, according also to the type of molecule in those two patient populations.

(a) **In cancer patients** with advanced disease (e.g., bone metastases or myeloma), suspension of antiresorptive drugs has to be decided on the basis of a difficult balance between a possible positive drug effect (reduction in the risk of Skeletal Related Events, SREs: bone fracture; need for radiotherapy; need for bone surgery; and spinal cord compression) and a potentially higher risk of a side effect anyway present (i.e., higher risk of MRONJ namely triggered by alveolar surgery). Clearly that balance could individually change on the basis of the moment along the patient cancer history and along the drug administration history: hypothetically, in the first months of drug administration for bone metastases or myeloma, the suspension is potentially more harmful for the (lack of reduction of) risk of SRE but less dangerous for the risk of triggering MRONJ; vice versa after two years of antiresorptive administration, for example, the suspension appears less dangerous for the risk of SRE (anyway present) whereas the risk of MRONJ (being higher with longer time and higher cumulative drug dose) might receive maximum benefit from the suspension.

Furthermore, the balance might change on the basis of the different characteristics of the antiresorptive drugs:

- BPs have a long half-life and are able to accumulate in the bone tissue, potentially influencing the post-extractive bone tissue repair. Furthermore, for some largely used BPs (above all zoledronic acid), a possible antiangiogenic effect was described in the literature, potentially reducing the soft tissue repair process. Consequently, a precautionary and temporary suspension of the drug administration has been claimed by most of authors as potentially useful, even if no definitive data have been published;
- Denosumab (Xgeva^®^), a monoclonal human anti-RANKL inhibitor, is a drug with a short half-life and does not accumulate in the bone. A possible rebound effect on the bone turnover has been described after drug withdrawal, so that some authors advised against a (long) suspension.

(b) **Osteometabolic patients**, cancer patients treated with antiresorptive agents for CTIBL or people affected by other non-malignant diseases (e.g., Paget disease), receive antiresorptive drugs at lower doses and less frequently (e.g., oral bisphosphonates; yearly 5 mg i.v. zoledronic acid; and bi-yearly 60 mg s.c. denosumab). The fracture risk can be determined objectively and correctly by applying the DeFRA79 algorithm validated by AIFA, the Italian version of the FRAX algorithm [44].

On the basis of the two different antiresorptive agents, they are classified as follows:

- BPs (e.g., alendronate, ibandronate) are synthetic analogues of pyrophosphates, which firmly bind to the hydroxyapatite and reduce bone metabolism/remodeling [6]. The half-life of BPs in circulation is short (ranging from 30 min to 2 h); however, once they have been incorporated into bone tissue, they can persist more than 10 years, depending on the skeletal turnover time [11,45]. Usually, BPs (i.e., alendronate) is administered orally (e.g., 150 mg/every 4 week); in a few patients BPs are administered intramuscularly (e.g., neridronate 2 mg/kg every 3 months) or intravenously (e.g., zoledronate 5 mg/every 12 months) [6,14,45].
- Denosumab (Prolia^®^), a monoclonal human IgG2 antibody that highly binds the receptor activator of nuclear factor-kB ligand (RANK-L), blocks the osteoclast maturation, function and survival. Its half-life is 25–32 days and it does not amass in the bone with a peculiar regimen (1 dose s.c. of 60 mg every six months) [46,47,48]. The suspension of denosumab determines within the following 3–6 months the rebound of the fracture risk, especially in patients at high risk of fracture [47,49,50]. Of note, when a patient is treated with denosumab, after a previous BP administration, he/she must be classified with the ONJ risk profile for the BP user.

#### 2.5.1. Questionable Practices

(#1) Performing dental extraction or oral surgery in patients with active cancer and myeloma bone disease and in therapy with i.v. high-dose bisphosphonates or denosumab (Xgeva^®^) without agreeing any temporary suspension with the prescriber (oncologist/hematologist).

(#2) Suspending indiscriminately therapy with antiresorptive drugs (i.e., bisphosphonates, denosumab) prescribed for osteoporosis (or its prevention), in case of necessity of extraction or programmed oral surgery, without agreeing with the prescribing physician.

#### 2.5.2. Good Practices

(#1) Plan, in cancer patients, combined assessment by the prescriber (i.e., high risk versus low risk of fracture and other SREs) and by the dentist (i.e., high risk versus low risk of post-extraction complications) to determine whether or not there is the need for a precautionary suspension of i.v. high-dose bisphosphonate or denosumab (Xgeva^®^) before and after the dental procedure. This combined assessment is mandatory in the absence of univocal data on the efficacy of the suspension of bisphosphonates or denosumab to reduce the risk of “post-extraction” MRONJ, and in consideration of conflicting data about the possible anti-angiogenic effect of zoledronic acid (Table 2) [51,52,53,54]. A suspension frequently applied consists of a period of 3 weeks, at least, from the last assumption before any elective invasive oral or dental procedures, and for the necessary waiting time until mucosal healing occurred before re-assumption (at least 4–6 weeks) (updated) [48,55,56].

(#2) Plan, in osteometabolic patients, a balanced and combined assessment by the prescriber (i.e., high risk versus low risk of fracture) and by the dentist (i.e., high risk versus low risk of post-extraction complications) to assess whether or not you need precautionary suspension of BPs or postponing the denosumab (Prolia^®^) *(updated)*. There are two different conditions (Table 2).

(a) Patient under BPs, a drug suspension may be already considered useful one week before surgical procedures. BP administration can resume once the biological process of healing of the oral tissues is completed (at least 4–6 weeks after surgery) [5,14];

(b) Patient under denosumab (Prolia^®^); it is still possible to take advantage of the pharmacokinetic of Prolia^®^ in order to define a time interval in those postponable and non-critical dental/periodontal conditions requiring invasive treatment and can theoretically take place without restrictions. This “delayed dosing window” lasts about 2 months, starts ideally 5 months after the last dose of Prolia^®^ and ends at the beginning of the 7th month. If the surgical dental procedure is assessed as urgent and not procrastinable, it is recommended to apply the suspension scheme of denosumab previously described for cancer patients [14,19].

### 2.6. MRONJ Therapy: Practices at Risk of Inappropriateness and Good Practices

Assume that the Consensus Conference embraced the principles and good practices promoted by the new recommendations by SIPMO/SICMF (June 2020) with respect to therapeutic strategies [6,14]; it focuses, here, on the following hot topics: perioperative antibiotic regimen; bone biopsy in patients suffering from clinical–radiological evidence of MRONJ; role of preoperative imaging; anticipated surgery versus planned exfoliation (self-sequestration); the shaving and smoothing of bone surfaces during surgical therapy; surgery-related quality of life issues for MRONJ patients; impact of the type of medication on the treatment decision-making of patients with BP-related and non-BP related (anti-RankL and/or targeted therapies) MRONJ; surgery for asymptomatic MRONJ; and the role of bone turnover markers for surgery.

#### 2.6.1. Questionable Practices

(#1) Forgetting the adoption of an adequate perioperative antibiotic regimen in case of surgical treatment of MRONJ.

(#2) Performing a diagnostic bone biopsy, unless bone metastases are suspected.

(#3) Applying of first-level imaging (e.g., dental X-ray and panoramic radiograph) only to plan surgical treatment.

(#4) Awaiting the exfoliation (self-sequestration) of necrotic exposed bone via the use of non-surgical therapies, since this process is unpredictable over time.

(#5) Neglecting shaving and smoothing of the bone surfaces as a mainstay of any surgical procedure.

(#6) Banishing the surgical option for MRONJ cancer patients based on general statements of residual life expectancy.

(#7) Merging BP-related and non-BP-related (anti-RankL and/or targeted therapies) MRONJ patients as a whole when considering temporary interruption of medications for surgical treatment.

(#8) Adopting surgical treatment of MRONJ in symptomatic cases only.

(#9) Relaying on systemic bone turnover markers to predict the success of surgical therapies in MRONJ patients.

#### 2.6.2. Good Practices

(#1) Prescribe a broad-spectrum antibiotic regimen as an integral part of surgical treatment: high-dose amoxicillin/clavulanic acid (1000 mg TID), plus high-dose Metronidazole (500 mg TID), from the day before surgery and up to the 10th post-operative day. Alternatives should be used in case of reported allergy to penicillin (confirmed).

(#2) Perform a diagnostic bone biopsy in case of clinical and radiological suspicion of bone metastases to the jaw or myeloma area, in order to distinguish them from site of MRONJ disease, when clinically needed (confirmed).

(#3) Use second-level imaging tools, mainly computed tomography (CT), to appropriately plan the extent of jawbone disease before surgery (confirmed).

(#4) Anticipate surgical treatment, whenever indicated, to reduce the surgical burden for MRONJ patients and increase the likelihood of long-term healing (confirmed).

(#5) Always perform the shaving and smoothing of bone surfaces as a mainstay of any surgical procedure to prevent further bone exposure (confirmed).

(#6) Plan the surgical treatment of MRONJ on an individual basis, weighing the impact and the potential benefit of surgery on the general health status of patients (confirmed).

(#7) Discuss and plan the temporary interruption of any given medication in agreement with the prescriber and based on its pharmacological properties, before initiating every MRONJ surgical treatment (confirmed).

(#8) Adopt the early surgical treatment also in MRONJ asymptomatic patients (confirmed).

(#9) Do not rely on systemic bone turnover markers to establish individual treatment algorithms for MRONJ, but carefully examine all potential factors that are likely to influence the long-term success of therapies, including the underlying disease (cancer or non-cancer) and the type of medication used (confirmed).

## 3. Conclusions

MRONJ is a potentially severe complication of antiresorptive and/or antiangiogenic treatment in patients with skeletal events due to various cancers as well as osteometabolic diseases; MRONJ may lead to a reduced quality of life due to jawbone infections, chronic pain, tooth loss and compromised function. Although notable progress has been made, there remain a number of controversial aspects on MRONJ, especially regarding pathogenesis, diagnosis and treatment.

The present paper updated conclusions from the Consensus Conference at the Symposium of Italian Society of Oral Pathology and Medicine (SIPMO) (20 October 2018, Ancona, Italy), after the newest recommendations on MRONJ were published in 2020 by two scientific societies (Italian Societies of Maxillofacial Surgery and Oral Pathology and Medicine, SICMF and SIPMO), written on the inputs of the experts of Italian Allied Committee on ONJ (IAC-ONJ). Some recommendations were confirmed.

We defined six issues of MRONJ and submitted for every issue the current knowledge and the appropriate best practices, in order to improve the management of patients.

We highlighted the importance to disclose the appropriate information about MRONJ, and the statements and recommendations presented in this paper might represent a beneficial instrument both at a national and international level to better understand and manage this singular and severe disease.

In the absence of clear knowledge on the etiology and given the continuous spread of the application of new target therapies, it will be fundamental to continue the research, to report any cases using new drugs and to clarify by evidence-based medicine all the controversial aspects of MRONJ.

## Figures and Tables

**Table 1:** Drug-related, systemic and local risk factors of Medication-Related Osteonecrosis of Jaws (MRONJ).

Risk Factor		
Drug-Related	Systemic	Local
Product (Antiresorptive/antiangiogenic drug) Route of administration (po, sc, iv, im) Cumulative dosage Duration of treatments Supportive care (e.g., chemotherapy, steroids, thalidomide)	Underlying disease (solid tumors, multiple myeloma, osteoporosis) Comorbidity (e.g., diabetes, rheumatoid arthritis, hypocalcemia, hyperparathyroidism) Lifestyle (e.g., smoking)	Dental/periodontal infection Peri-implantitis Oral surgeries (e.g., dental extractions) Unfitting removable dentures Anatomical conditions (e.g., torus, exostosis, pronounced mylohyoid ridge)

**Table 2:** Drug suspensions proposed for cancer and osteometabolic patients [14,19].

**Drug Suspensions in Cancer Patients**		
**Active Pharmaceutical Ingredient**	**Last Administrations before Surgical Procedure**	**Resume Treatment**
Bisphosphonates	At least 1 week before	At least 4–6 weeks after surgical procedures
Denosumab (Xgeva^®^)	At least 3 weeks before	
Bevacizumab	At least 5–8 weeks before	
Sunitinib	At least 1 week before	
**Drug Suspensions in Osteometabolic Patients**		
**Active Pharmaceutical Ingredient**	**Last Administrations before Surgical Procedure**	**Resume Treatment**
Bisphosphonates *	At least 1 week before	At least 4–6 weeks after surgical procedures
Denosumab (Prolia^®^)	No suspension **	

* Only in patients exposed to BPs for more than 3 years or in patients exposed to BPs for less than 3 years but in the presence of other systemic or local risk factors (e.g., concomitant use of corticosteroids, diabetes, and rheumatoid arthritis). ** It is still possible to maximize the pharmacokinetic of Prolia^®^ and identify a time interval in those postponable and non-critical dental/periodontal conditions requiring invasive treatment, and can ideally take place without restrictions. This “delayed dosing window” lasts about 2 months, starts ideally 5 months after the last dose of Prolia^®^ and ends at the beginning of the 7th month.
